# The NEUROSAVE study – impact of cadaveric neurosurgical workshops on general and paediatric surgeons’ confidence in performing life-saving neurosurgical procedures: a preliminary survey study

**DOI:** 10.1186/s12909-026-08993-3

**Published:** 2026-03-12

**Authors:** Wojciech Górecki, Michał Szymoniuk, Łukasz Domagalski, Jakub Litak, Grzegorz Turek, Klaudia Kus-Budzynska, Jacek Kunicki, Paweł Szmygin, Paweł Nachulewicz, Kamila Ćwik, Kami Torres, Jacek Baj, Grzegorz Staśkiewicz, Wojciech Czyżewski

**Affiliations:** 1 Department of Neurosurgery, The National Institute of Medicine of the Ministry of Interior and Administration, Warsaw, Poland; 2 Department of Neurosurgery, Medical University of Lublin, Jaczewskiego 8, Lublin, 20-090 Poland; 3 Department of Clinical Immunology, Medical University of Lublin, Chodźki 4a, Lublin, 20-093 Poland; 4 General Surgery Department with Neurosurgery Subunit, Poviat Specialist Hospital in Stalowa Wola, ul. Staszica 4, Stalowa Wola, 37-450 Poland; 5 Department of Neurosurgery, Brodno Masovian Hospital, Warsaw, Poland; 6 Department of Neurosurgery, Maria Sklodowska-Curie National Research Institute of Oncology, ul. W.K. Roentgena 5, Warsaw, 02-781 Poland; 7 The Department of Pediatric Surgery and Traumatology, University Children’s Hospital of Lublin, Medical University of Lublin, Lublin, Poland; 8 Laboratory of Management Education in Health Care, Faculty of Medicine, Medical University of Lublin, Lublin, Poland; 9 Department of Plastic, Reconstructive Surgery with Microsurgery, Medical University of Lublin, Jaczewskiego 8, Lublin, 20-090 Poland; 10 Department of Correct, Clinical and Imaging Anatomy, Medical University of Lublin, Jaczewskiego 4, Lublin, 20-090 Poland; 11 Department of Didactics and Medical Simulation, Medical University of Lublin, Chodźki 4, Lublin, 20-093 Poland; 12 1st Department of Radiology, Medical University of Lublin, Jaczewskiego 8, Lublin, 20-090 Poland

**Keywords:** Cadaveric simulation, Neurosurgical emergencies, Confidence, General surgery, Pediatric surgery, Trauma, Education

## Abstract

**Background:**

In many European settings, general and pediatric surgeons may be the first and sometimes the only physicians available to perform life‑saving neurosurgical procedures in emergency situations. However, especially in Poland, contact with cranial trauma surgery during training is limited. We evaluated whether a cadaveric, neurosurgeon‑led workshop (NEUROSAVE) improves participants’ confidence in essential procedures.

**Methods:**

We conducted a prospective survey study during a cadaveric neurosurgical workshop (*n* = 15). Participants assessed their level of confidence (on a Likert scale from 1 to 5) in relation to individual stages of the procedures performed (e.g. burr holes, craniotomies, decompressive craniectomy) and emergency operations (e.g. evacuation of epidural, acute and chronic subdural, and intraparenchymal haematomas) immediately before and after the course; a subset provided last follow‑up ratings. Data are presented as medians (IQR); paired Wilcoxon tests assessed pre‑post and baseline‑follow‑up differences.

**Results:**

Median confidence increased significantly immediately after the course across core skills, including Mayfield fixation (1.00→2.00; *p* = 0.01), burr‑hole drilling (2.00→3.00; *p* = 0.01), frontal craniotomy (1.00→2.00; *p* = 0.02), temporal craniotomy (2.00→3.00; *p* = 0.03), decompressive craniectomy (1.00→2.00; *p* = 0.01), duroplasty (2.00→3.00; *p* = 0.01), dural sealing (1.00→2.00; *p* = 0.02), bone‑flap restoration (2.00→3.00; *p* < 0.01) and evacuations of EDH (1.00→2.00; *p* = 0.01), acute SDH (1.00→2.00; *p* = 0.02) and ICH (1.00→2.00; *p* = 0.01). Chronic SDH showed a non‑significant trend (*p* = 0.09). At the last follow‑up (*n* = 7), point estimates suggested retention in selected domains, albeit with small sample size and variable exposure to practice.

**Conclusions:**

Short neurosurgical workshops on cadavers significantly improve the self-confidence of general and pediatric surgeons in performing life‑saving cranial trauma procedures. Such interdisciplinary, simulation‑based programmes could address urgent training gaps in systems where neurosurgical expertise may be unavailable on‑site, particularly in regional hospitals in Poland. Further multicenter studies with objective outcome assessment are needed. The present analysis was based on the first educational cycle of the NeuroTrauma Days, a new interdisciplinary simulation program established in Lublin in 2025. This inaugural edition served as a pilot evaluation of its educational impact and feasibility among general and pediatric surgeons. Given the encouraging preliminary outcomes, we plan to continue and expand the NeuroTrauma Days series, integrating additional modules (e.g. spine and peripheral nerve trauma) and implementing longitudinal assessments combining self-confidence metrics with objective performance evaluation (e.g. OSATS-based scoring and case-log audits). Such follow-up studies will allow for a more comprehensive understanding of skill retention, translation to clinical practice, and the broader role of cadaveric simulation in surgical education across disciplines.

**Supplementary Information:**

The online version contains supplementary material available at 10.1186/s12909-026-08993-3.

## Introduction

General and pediatric surgeons working in regional or district hospitals may encounter emergency time-critical neurotrauma, including epidural and acute subdural hematomas, in which immediate surgical intervention can save lives. In practice, outside of neurosurgical centers, formal exposure to cranial trauma procedures as part of basic training is rare. Poland exemplifies this gap: the availability of on‑site neurosurgical coverage varies, with many surgeons reporting minimal practical experience with burr holes, decompressive craniectomy, or dural repair before entering independent practice. Similar gaps in emergency neurosurgical training for non-neurosurgeons have been reported across Europe and Asia, underscoring the global relevance of structured cadaveric programmes. Simulation‑based education, including cadaveric training, has been proposed to mitigate such deficits by providing realistic, supervised rehearsal of operative steps in a risk‑free environment. Building on international initiatives ranging from the ATLS framework for early trauma management [1, 2] to European programmes that integrate simulation with cadaver dissection [3] we designed the NEUROSAVE workshop to equip non‑neurosurgeons with practical skills for life‑saving cranial procedures.

This study describes changes in self-rated confidence in key stages and rescue operations before the course, after the course, and during the observation period. We hypothesized that a single, structured workshop using human cadavers would result in an immediate increase in confidence, which would be partially maintained during the observation period. This study aimed to evaluate the educational and surgical impact of the NeuroSave cadaveric training program using structured participant feedback.

In health systems with reliable rapid access to neurosurgical centres, the preferred pathway for most acute intracranial lesions is early transfer and specialist management. However, in many regional settings, timely transfer is not consistently achievable, and the clinical course of selected lesions can deteriorate within minutes to hours. NEUROSAVE was therefore designed as an adjunct to established trauma pathways, not a replacement for neurosurgical care. Its purpose is to provide supervised, protocol-oriented rehearsal of time-critical, life-saving cranial steps that may be required as a temporising ‘damage-control’ intervention when on-site neurosurgical coverage is unavailable and transfer would be delayed beyond a clinically acceptable window.

## Materials and methods

### Study design and setting

We conducted a prospective survey study as part of neurosurgical workshops using cadavers, intended for general and pediatric surgeons. Participants completed anonymous paper questionnaires immediately before and after the course; some of them provided answers during the final check-up. The study formed part of routine educational activities within the institutional simulation/didactics programme. As the intervention represented standard educational practice and questionnaires were anonymised and voluntary, formal ethics committee approval was not required (see Ethics).

### Course description

The curriculum covered patient positioning and Mayfield frame fixation; burr‑hole drilling; frontal and temporal craniotomy; suboccipital craniectomy; decompressive craniectomy; dural detachment; subdural drain implantation; duroplasty (including adjuncts such as TachoSil); Dandy and Poppen sutures; dural sealing (e.g. polymer sealant); and bone‑flap restoration. Emergency procedures included evacuations of epidural, acute and chronic subdural, and intraparenchymal hematomas. The course consisted of a one-day (8-hour) hands-on training session with six cadaveric stations supervised by specialist neurosurgeons. Faculty (consultant neurosurgeons) led each station with step‑wise demonstration, deliberate practice, and structured feedback.

### Participants

Fifteen surgeons (general surgery *n* = 2, pediatric surgery *n* = 13) attended and completed pre/post questionnaires; seven provided last follow‑up data. Professional seniority included residents and board‑certified specialists. Prior exposure to head‑trauma surgery varied from none to > 50 assisted cases; most had limited independent operating experience in cranial trauma.

### Survey and outcomes

The primary outcome was self‑reported confidence in procedural steps and emergency operations, rated on a 5‑point Likert scale (1 = not confident, 5 = highly confident). Items were identical at baseline, immediately post‑course, and at the last follow‑up (with a ‘not performed’ option at follow‑up). The questionnaire was adapted from previously published cadaveric training evaluations and refined by an expert panel of neurosurgeons for content validity [4]. 

### Statistical analysis

Continuous variables are reported as medians with interquartile ranges (IQR). Categorical data are presented as counts and percentages. Pre‑ to post‑course and baseline‑to‑follow‑up comparisons used paired Wilcoxon tests (two‑sided; α = 0.05). Analyses were performed in R 4.5.1.

## Results

### Characteristics of study participants

The workshop hosted 19 participants. Of them, a total of 15 participants, with a mean age of 40.40 ± 12.48 years, completed the questionnaire evaluating the confidence in surgical techniques and procedures (Table 1). Female participants constituted 33.3% of the sample (*n* = 5). Regarding professional status, the group included 7 residents (46.7%) and 8 specialists (53.3%). In terms of academic titles, the majority of participants held an MD degree (9 participants, 60.0%). Additionally, 4 participants (26.7%) held a PhD, while 1 participant (6.7%) had a DSc, and 1 participant (6.7%) held the title of professor. Most participants were pediatric surgeons (13 participants, 86.7%), while 2 participants (13.3%) were general surgeons. Most had assisted in ≤ 10 cranial trauma cases; none had independently performed > 10 cases.

**Table 1:** Characteristics of the studied population

Characteristic	*N* = 15^*1*^
Age (years)	40.40 (12.48)
Gender	
female	5 (33.3%)
male	10 (66.7%)
Professional status	
resident	7 (46.7%)
specialist	8 (53.3%)
Scientific title	
MD	9 (60.0%)
PhD	4 (26.7%)
DSc	1 (6.7%)
Professor	1 (6.7%)
Speciality or residency	
general surgeon	2 (13.3%)
pediatric surgeon	13 (86.7%)
Participation in head trauma surgeries	13 (86.7%)
Participation as assistant	
did not participate	2 (13.3%)
less than 10 times	9 (60.0%)
between 11 and 50 times	3 (20.0%)
between 51 and 100 times	1 (6.7%)
more than 100 times	0 (0.0%)
Participation as lead surgeon	
did not participate	9 (60.0%)
less than 10 times	6 (40.0%)
between 11 and 50 times	0 (0.0%)
between 51 and 100 times	0 (0.0%)
more than 100 times	0 (0.0%)

^*1*^ Mean (SD); *n* (%)

A large proportion of the group (13 participants, 86.7%) reported participation in head trauma surgeries, indicating substantial clinical experience in this field. Considering.

participation as a surgical assistant, the majority (9 participants, 60.0%) had taken part in fewer than 10 surgeries. Three participants (20.0%) had assisted in 11–50 procedures, and one (6.7%) in 51–100 surgeries. Two participants (13.3%) had not participated as assistants, and none had assisted in more than 100 surgeries. Regarding participation as the lead surgeon, 9 participants (60.0%) reported no experience in this role, while 6 participants (40.0%) had performed fewer than 10 surgeries as lead surgeons. None of the participants had led more than 10 surgical procedures.

### Pre‑ to post‑course changes in confidence

**Fig. 1:**
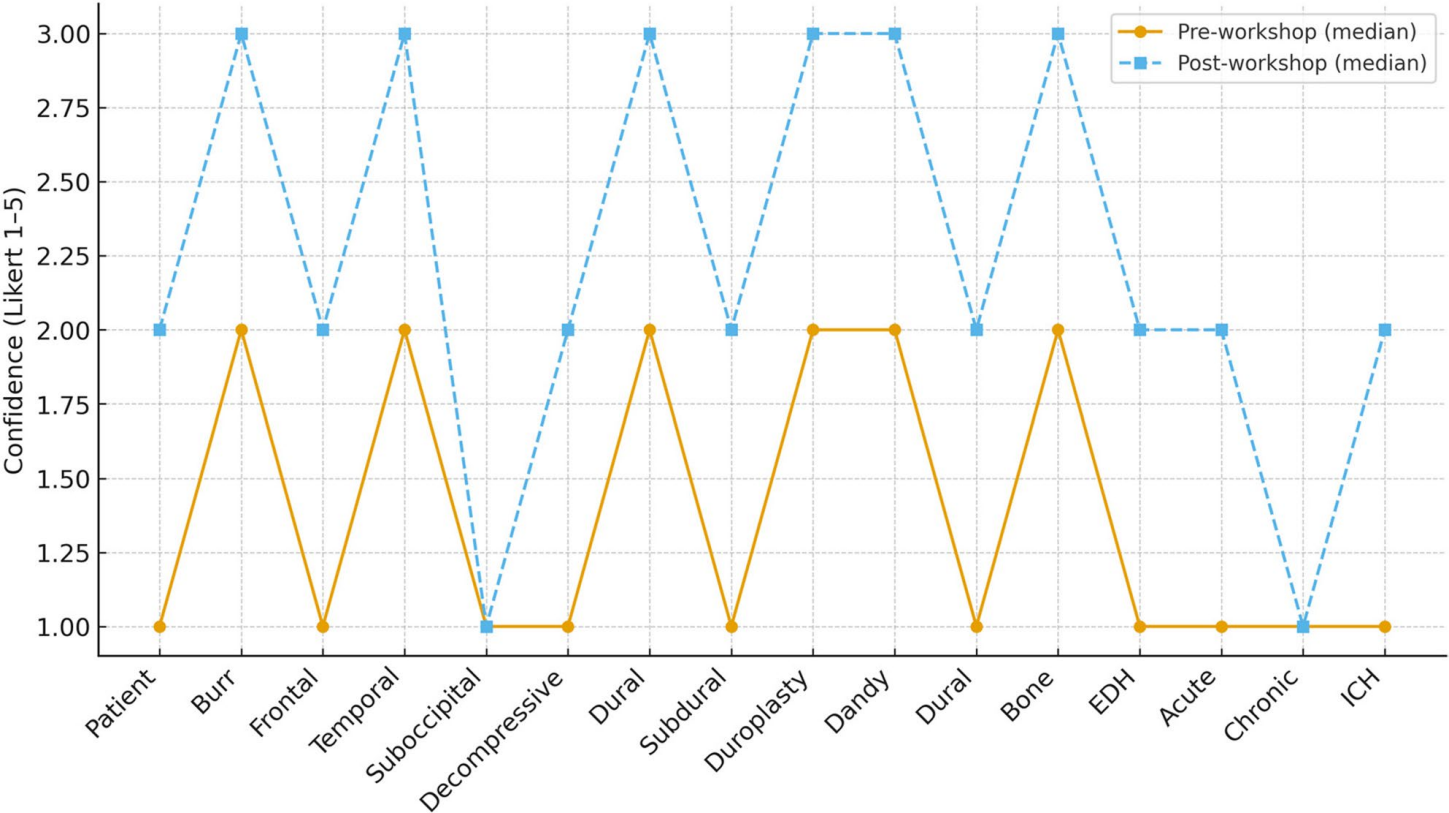
Median confidence before and after the course (*n* = 15)

Confidence improved immediately after training across most procedural steps (Table 2; Fig. 1). Significant improvements (all *p* ≤ 0.03) were observed for Mayfield fixation, burr‑hole drilling, frontal and temporal craniotomy, decompressive craniectomy, dural detachment, duroplasty, Dandy‑Poppen sutures, dural sealing, bone‑flap restoration, and evacuations of epidural, acute subdural, and intraparenchymal hematomas. Suboccipital craniectomy and subdural drain implantation showed borderline results *p* = 0.05; chronic subdural evacuation trended towards improvement (*p* = 0.09).

**Table 2:** Confidence before and after the course (Likert 1–5); values are medians (IQR); Wilcoxon paired tests, *n* = 15

Surgical skill	Confidence in Likert scale		*p*-value²
	Pre-workshop^*1*^ *n* = 15	Post-workshop^*1*^ *n* = 15	
Patient positioning and Mayfield frame fixation	1.00 (1.50)	2.00 (2.00)	0.01
Burr hole drilling	2.00 (1.50)	3.00 (3.00)	0.01
Frontal craniotomy	1.00 (1.00)	2.00 (2.00)	0.02
Temporal craniotomy	2.00 (2.00)	3.00 (1.50)	0.03
Suboccipital craniectomy	1.00 (0.00)	1.00 (1.50)	0.05
Decompressive craniectomy	1.00 (0.00)	2.00 (1.00)	0.01
Dural detachment	2.00 (2.00)	3.00 (2.00)	0.02
Subdural drain implantation	1.00 (1.50)	2.00 (2.50)	0.05
Duroplasty	2.00 (2.00)	3.00 (1.50)	0.01
Dandy and Poppen sutures	2.00 (2.50)	3.00 (2.00)	0.01
Dural sealing	1.00 (1.00)	2.00 (1.50)	0.02
Bone flap restoration	2.00 (2.50)	3.00 (2.00)	< 0.01
EDH evacuation	1.00 (1.00)	2.00 (2.00)	0.01
Acute SDH evacuation	1.00 (1.00)	2.00 (2.00)	0.02
Chronic SDH evacuation	1.00 (1.50)	1.00 (2.50)	0.09
ICH evacuation	1.00 (0.00)	2.00 (1.00)	0.01

*EDH* epidural hematoma, *SDH* subdural hematoma, *ICH* intracerebral hematoma

1 - median (IQR), 2 - paired Wilcoxon test

### Follow‑up

**Fig. 2:**
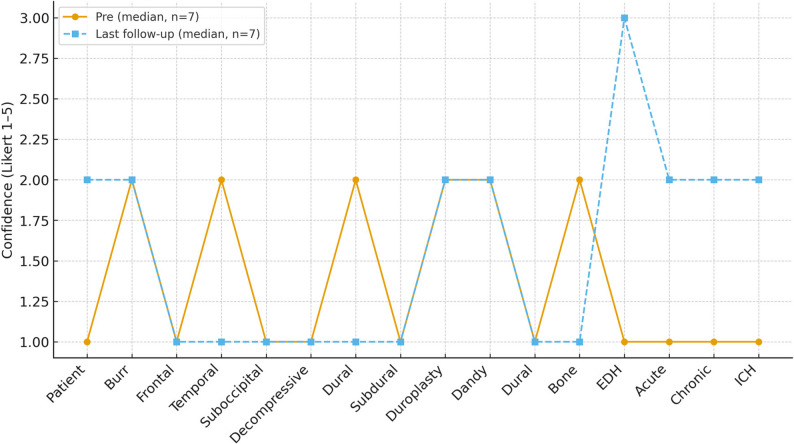
Median confidence at baseline and last follow‑up (*n* = 7)

Seven participants returned the last follow‑up questionnaire. Median confidence remained at or above baseline in several domains (Table 3; Fig. 2), though sample size was small and clinical exposure heterogeneous. Some technical steps showed limited retention without ongoing practice or supervised exposure, underscoring the need for refresher sessions and integration with in‑hospital mentorship.

**Table 3:** Confidence at baseline and last follow‑up (Likert 1–5); medians (IQR); paired Wilcoxon signed-rank test, *n* = 7

Surgical skill	Confidence in Likert scale		*p*-value^2^
	Pre-workshop^*1*^ *n* = 7	At last follow-up^*1*^ *n* = 7	
Patient positioning and Mayfield frame fixation	1.00 (0.50)	2.00 (2.00)	0.15
Burr hole drilling	2.00 (1.00)	2.00 (2.00)	0.16
Frontal craniotomy	1.00 (0.50)	1.00 (1.50)	0.08
Temporal craniotomy	2.00 (1.00)	1.00 (1.00)	0.02
Suboccipital craniectomy	1.00 (0.00)	1.00 (0.00)	0.02
Decompressive craniectomy	1.00 (0.00)	1.00 (1.00)	0.15
Dural detachment	2.00 (1.00)	1.00 (1.50)	0.04
Subdural drain implantation	1.00 (1.00)	1.00 (2.00)	0.19
Duroplasty	2.00 (1.00)	2.00 (2.50)	0.12
Dandy and Poppen sutures	2.00 (1.00)	2.00 (0.50)	0.05
Dural sealing	1.00 (0.50)	1.00 (1.00)	0.09
Bone flap restoration	2.00 (1.00)	1.00 (1.50)	0.02
EDH evacuation	1.00 (0.50)	3.00 (2.00)	0.69
Acute SDH evacuation	1.00 (0.50)	2.00 (1.50)	0.38
Chronic SDH evacuation	1.00 (0.50)	2.00 (1.00)	0.13
ICH evacuation	1.00 (0.00)	2.00 (1.00)	0.28

*EDH* epidural hematoma, *SDH* subdural hematoma, *ICH* intracerebral hematoma

^*1*^ Median (IQR)

^2^ Wilcoxon rank sum test EDH - epidural hematoma, SDH - subdural hematoma, ICH - intracerebral hematoma

## Discussion

The limited sample size reflects the pilot character of the programme and serves primarily to inform the design of a multicentre study currently in preparation. In this preliminary study, a focused cadaveric workshop delivered immediate, statistically significant gains in self‑reported confidence across most steps of life‑saving cranial trauma surgery among general and pediatric surgeons. While confidence may not directly equate to competence, self-efficacy has been associated with engagement and performance in procedural training [5]. These findings align with international reports that cadaveric simulation enhances confidence and perceived readiness [6–9]. The magnitude and breadth of improvements across core steps - from Mayfield fixation and burr‑holes to decompressive craniectomy and dural repair - support the face and content validity of the curriculum.

From a systems perspective, transfer to a neurosurgical centre remains the preferred pathway for most acute intracranial lesions when it can be achieved rapidly. The rationale for NEUROSAVE is limited to scenarios in which timely neurosurgical coverage or transfer is not feasible, and where deterioration can occur within a narrow time window. In such circumstances, a protocolised temporising intervention may serve as a bridge to definitive specialist care rather than an alternative to it [10]. 

Our data address a specific systems‑level need: outside neurosurgical centres, surgeons may have to perform time‑critical procedures before specialist transfer is feasible. Frameworks such as ATLS provide a shared language and early management principles [1, 2], and updated ACS best‑practice guidelines for traumatic brain injury emphasise rapid recognition and timely intervention pathways [10]. Yet, neither can substitute for hands‑on rehearsal of the operative steps required when neurosurgical cover is unavailable or delayed [10]. At the same time, interhospital transfer patterns show that a substantial proportion of transferred TBI patients ultimately do not undergo neurosurgical intervention, highlighting the importance of triage, clear indications, and governance, rather than indiscriminate escalation [11]. Against this background, NEUROSAVE is best understood as a structured educational intervention that prepares non-neurosurgeons to execute strictly bounded temporising steps within an explicit transfer-first system, not to expand the scope of independent operative practice.

The included procedures span lesions with different urgency profiles, including EDH, acute SDH, and intraparenchymal haemorrhage, and these entities should not be treated as equivalent with respect to the rationale for local intervention. For EDH and acute SDH, existing surgical guidance emphasises prompt decompression and specialist pathways, reflecting the time-dependent risk of deterioration and the priority of rapid access to neurosurgical care [12]. For acute SDH specifically, classic outcome data demonstrate a strong association between delays to surgery and worse outcomes, reinforcing that temporising action should be reserved for exceptional “in extremis” situations when timely transfer is not feasible [13]. In contrast, intraparenchymal haemorrhage is often urgent rather than emergent, and randomised evidence supports careful patient selection and specialist decision-making, without a consistent broad benefit for early surgery across unselected patients [14]. Notably, the STICH trials primarily evaluated spontaneous supratentorial ICH rather than traumatic intraparenchymal haemorrhage; we cite them here to emphasise the need for careful patient selection and specialist decision-making in lesions that are often urgent rather than immediately life-threatening, which supports a transfer-first approach. For this reason, the programme should be interpreted as rehearsal of a technical sequence within a governed pathway, where EDH and selected SDH scenarios represent the clearest time-critical justification, while ICH exposure is primarily educational and any real-world application should remain highly restricted and protocol-defined.

Table 4 summarizes the intended scope and governance of NEUROSAVE within a transfer-first system. In brief, EDH with neurological deterioration is mapped to a time-critical scenario where immediate temporising decompression may be considered only when timely transfer is not feasible; acute SDH is treated as a rare “in extremis” exception under similarly restrictive conditions; and intraparenchymal hemorrhage is presented primarily as educational exposure, with transfer and specialist decision-making remaining the default. This explicit stratification is intended to prevent overinterpretation of the curriculum as endorsing broad independent operative management by non-neurosurgeons and to anchor any potential clinical application within predefined indications, neurosurgical consultation, and audit. Importantly, this pilot assessed educational outcomes (self-reported confidence) rather than objective technical performance or patient outcomes; any implementation should therefore remain protocol-bound, transfer-first, and embedded within defined consultation and audit processes.

**Table 4:** Intended clinical role and governance of procedures included in NEUROSAVE (conceptual framework)

Procedure / pathology	Typical time-criticality	Intended role for non-neurosurgeon in a regional hospital	Minimal prerequisites (examples)	Transfer principle
EDH (evacuation)	Emergent	Temporising life-saving decompression/evacuation when neurological deterioration and transfer delay	CT-confirmed EDH with mass effect; deterioration (GCS drop, anisocoria); neurosurgical support unavailable; transfer delay expected	Transfer to neurosurgical centre as soon as feasible after temporising step
Acute SDH (decompression)	Emergent in selected cases	Temporising decompressive craniectomy or limited evacuation only in extremis when transfer not feasible in time	CT-confirmed ASDH with significant mass effect; rapid deterioration; no timely transfer	Transfer after stabilisation; definitive care at centre
ICH (evacuation)	Often urgent rather than emergent	Primarily educational exposure; clinical implementation should be highly restricted and protocol-bound	Clear neurosurgical indication and inability to transfer in a clinically acceptable timeframe	Default is transfer; local operative intervention only in exceptional, protocol-defined scenarios
Burr holes, craniotomies, duroplasty, haemostasis steps	Supportive technical steps	Technical sequence rehearsal to enable safe execution of a temporising procedure under supervision/tele-mentoring	Cadaveric training, checklist, defined instrumentation, supervision plan	Always linked to a broader pathway and audit

Internationally, hybrid models combining simulation with cadaver dissection have been successfully implemented. The EANS Basic Brain Course integrates task trainers and cadaver labs and has been reported as a modern concept for neurosurgical training [3]. Systematic reviews consistently suggest that simulation in neurosurgery improves knowledge and technical performance, with cadaveric models offering superior anatomical realism [8, 15]. Recent low‑cost, regional neurosurgery bootcamps demonstrate feasibility, effectiveness, and scalable costs, particularly in resource‑constrained settings [16]. Our findings add to this literature by focusing on non-neurosurgeons, namely general and pediatric surgeons, who may be required to contribute to time-critical cranial pathways in regional emergency contexts. In that context, the key contribution of this pilot is not to argue for broader non-specialist operative management, but to show that a structured cadaveric course can rapidly increase confidence across a defined set of technical steps that may be needed as a temporising measure until definitive specialist care is reached.

Retention at follow‑up appeared variable, likely reflecting limited subsequent case exposure and the small follow‑up cohort (*n* = 7). This suggests that a longitudinal training pathway e.g. periodic refreshers, in‑theatre mentorship, and access to task trainers may be necessary to consolidate skills. Programmes that longitudinally integrate simulation have shown sustained confidence gains over several years [7].

Finally, regarding certification, while cranial exposure procedures may appear within some general surgery training curricula, this does not equate to a harmonised, procedure-specific credentialing pathway for independent emergency cranial trauma surgery, with defined indications, minimum case volumes, objective assessment standards, and maintenance-of-competence requirements across European high-income settings. We therefore propose a measurable competency framework for future implementation studies, including objective skills assessment (for example OSATS-based), supervised initial clinical cases, periodic re-certification, and ongoing audit of indications and outcomes. This approach aligns the educational intent of NEUROSAVE with governance and patient-safety requirements, and provides a transparent structure for evaluation in a prospective multicentre setting.

## Conclusions

The importance of additional training is particularly necessary in Poland, where, in theory, life-saving procedures that are the domain of neurosurgery are included in the general and pediatric surgery specialization program, but unfortunately, in reality, the possibility of performing them is very limited. The prevalence of general and pediatric surgery departments in Poland is also greater, meaning that with reduced access to neurosurgeons, situations requiring life-saving procedures will arise, making developing hematoma surgery skills even more crucial. Improving emergency neurosurgical capability among general surgeons can directly reduce preventable mortality from cranial trauma in regions lacking on-site neurosurgeons.

Cadaveric, neurosurgeon‑led simulation (NEUROSAVE) produced substantial immediate improvements in self‑reported confidence across key life‑saving cranial procedures among general and pediatric surgeons, addressing a critical training gap in systems where neurosurgical expertise may be inaccessible at the point of care. Scalable, interdisciplinary simulation programs, combined with periodic refresher training and mentoring, deserve wider implementation.

## Supplementary Information

Supplementary Material 1.

## Data Availability

The datasets generated and analysed during the current study are available from the corresponding author on reasonable request.

## Abbreviations

ATLS: Advanced Trauma Life Support
CT: Computed Tomography
EDH: Epidural Hematoma
EANS: European Association of Neurosurgical Societies
GCS: Glasgow Coma Scale
ICH: Intracerebral Hemorrhage
IQR: Interquartile Range
MD: Doctor of Medicine
NEUROSAVE: Neurosurgical Emergency Skills Acquisition Via Education
OSATS: Objective Structured Assessment of Technical Skills
SD: Standard Deviation
SDH: Subdural Hematoma
TBI: Traumatic Brain Injury
IQR: Interquartile Range
SD: Standard Deviation
