# Supplementary material for: The NEUROSAVE study – impact of cadaveric neurosurgical workshops on general and paediatric surgeons’ confidence in performing life-saving neurosurgical procedures: a preliminary survey study
Source: BMC Med Educ. 2026 Mar 12;26:640. doi: 10.1186/s12909-026-08993-3 (PMC13094141; doi:10.1186/s12909-026-08993-3)
Supplement: Supplementary file 1 — Supplementary Material 1. [file 12909_2026_8993_MOESM1_ESM.pdf]

Age:

Sex:

Employment status: resident/specialist

Academic degree:

Medical specialty:

A. Previous surgical experience in traumatic brain injury procedures

1. Have you previously actively participated in surgical procedures related to traumatic brain injuries?

Yes/No

2. How many times before participating in the workshop did you actively assist in surgical procedures related to traumatic brain injuries?

Did not participate

Less than 10 times

Between 11 and 50 times

Between 51 and 100 times

More than 100 times

3. How many times before participating in the workshop did you actively perform surgical procedures related to traumatic brain injuries as the primary surgeon?

Did not participate

Less than 10 times

Between 11 and 50 times

Between 51 and 100 times

More than 100 times

B. Confidence in performing procedures used in the surgical treatment of traumatic brain injuries

1. What was your level of confidence in performing the procedures listed below before participating in the workshop?

Please give your answer on a 5-point scale, where 1 = no confidence, 5 = high confidence

Procedure	No confidence				High confidence
	1	2	3	4	5
Patient positioning and fixation in the Mayfield frame					
Burr-hole drilling					
Frontal craniotomy					
Temporal craniotomy					
Suboccipital craniectomy					
Decompressive craniectomy with middle cranial fossa decompression					
Dura mater dissection					
Subdural drain implantation					
Dural plasty using Tachosil					
Placement of Dandy’s and Poppen’s sutures					
Sealing of the dura mater with Adherus					
Bone flap replacement					

2. What was your level of confidence in performing the procedures listed below immediately after completing the workshop?

Please give your answer on a 5-point scale, where 1 = no confidence, 5 = high confidence

Procedure	No confidence				High confidence
	1	2	3	4	5
Patient positioning and fixation in the Mayfield frame					
Burr-hole drilling					
Frontal craniotomy					
Temporal craniotomy					
Suboccipital craniectomy					
Decompressive craniectomy with middle cranial fossa decompression					
Dura mater dissection					
Subdural drain implantation					
Dural plasty using Tachosil					
Placement of Dandy’s and Poppen’s sutures					
Sealing of the dura mater with Adherus					
Bone flap replacement					

3. What is your level of confidence in performing the procedures listed below in clinical practice from the end of the workshop until now?

Please give your answer on a 5-point scale, where 1 = no confidence, 5 = high confidence, or mark "have not performed".

Procedure	No confidence				High confidenc e	Have not performed
	1	2	3	4	5	
Patient positioning and fixation in the Mayfield frame						
Burr-hole drilling						
Frontal craniotomy						
Temporal craniotomy						
Suboccipital craniectomy						
Decompressive craniectomy with middle cranial fossa decompression						
Dura mater dissection						
Subdural drain implantation						
Dural plasty using Tachosil						
Placement of Dandy’s and Poppen’s sutures						
Sealing of the dura mater with Adherus						

Bone flap replacement						

C. <u>**Confidence in performing surgical procedures related to traumatic brain injuries**</u>

1. **What was your level of confidence in performing the following surgical procedures before participating in the workshop?**

Please give your answer on a 5-point scale, where 1 = no confidence, 5 = high confidence

Procedure	No confidence				High confidenc e
	1	2	3	4	5
EDH evacuation					
Acute SDH evacuation					
Chronic SDH evacuation					
ICH evacuation					

2. What was your level of confidence in performing the following surgical procedures immediately after completing the workshop?

Please give your answer on a 5-point scale, where 1 = no confidence, 5 = high confidence

Procedure	No confidence		High confidence

	1	2	3	4	5
EDH evacuation					
Acute SDH evacuation					
Chronic SDH evacuation					
ICH evacuation					

3. What is your level of confidence in performing the following surgical procedures in clinical practice from the end of the workshop until now?

Please give your answer on a 5-point scale, where 1 = no confidence, 5 = high confidence, or mark „have not performed"

Procedure	No confidence				High confidence	Have not performed
	1	2	3	4	5	
EDH evacuation						
Acute SDH evacuation						
Chronic SDH evacuation						
ICH evacuation						
